# Frequency Clustering Analysis for Resting State Functional Magnetic Resonance Imaging Based on Hilbert-Huang Transform

**DOI:** 10.3389/fnhum.2017.00061

**Published:** 2017-02-16

**Authors:** Xia Wu, Tong Wu, Chenghua Liu, Xiaotong Wen, Li Yao

**Affiliations:** ^1^College of Information Science and Technology, Beijing Normal UniversityBeijing, China; ^2^Department of Psychology, Renmin University of ChinaBeijing, China

**Keywords:** fMRI, HHT, frequency, clustering, IMF

## Abstract

**Objective:** Exploring resting-state functional networks using functional magnetic resonance imaging (fMRI) is a hot topic in the field of brain functions. Previous studies suggested that the frequency dependence between blood oxygen level dependent (BOLD) signals may convey meaningful information regarding interactions between brain regions.

**Methods:** In this article, we introduced a novel frequency clustering analysis method based on Hilbert-Huang Transform (HHT) and a label-replacement procedure. First, the time series from multiple predefined regions of interest (ROIs) were extracted. Second, each time series was decomposed into several intrinsic mode functions (IMFs) by using HHT. Third, the improved k-means clustering method using a label-replacement method was applied to the data of each subject to classify the ROIs into different classes.

**Results:** Two independent resting-state fMRI dataset of healthy subjects were analyzed to test the efficacy of method. The results show almost identical clusters when applied to different runs of a dataset or to different datasets, indicating a stable performance of our framework.

**Conclusions and Significance:** Our framework provided a novel measure for functional segregation of the brain according to time-frequency characteristics of resting state BOLD activities.

## Introduction

Exploring resting-state functional networks using functional magnetic resonance imaging (fMRI) is a persistent topic in the research field of brain functions (Raichle et al., 2001; Damoiseaux et al., 2006; De Luca et al., 2006). From a perspective of examining the features of the signal, the conventional functional network analysis methods may fall into two categories: (1) the time-based methods, such as temporal correlation (Fox et al., 2005; Fransson and Marrelec, 2008; Lowe, 2010; Van Den Heuvel and Pol, 2010), regional homogeneity (ReHo) (Zang et al., 2004), independent analysis method (ICA) (De Luca et al., 2006; Calhoun et al., 2009), and Bayesian network analysis (Li et al., 2011; Wu et al., 2011), and (2) the frequency-based methods, such as low-frequency fluctuation (ALFF) analysis (Yang et al., 2007), coherence analysis (Salvador et al., 2005), total interdependence analysis (Wen et al., 2012), and phase relationship analysis (Sun et al., 2005).

The time-based methods are usually convenient and effective in examining the point-to-point relationship between regional blood oxygen level dependent (BOLD) signals. Recent studies suggested that the frequency dependence between BOLD signals may also convey meaningful information regarding interactions between brain regions (Wen et al., 2012; Yu et al., 2013; Wei et al., 2014). In a recent fMRI study (Song et al., 2014), a ReHo based frequency clustering analysis framework was introduced for resting-state fMRI analysis. The BOLD time series of each voxel was decomposed into several frequency components using empirical mode decomposition (EMD), and the ReHo values of the components were used as features for clustering the voxels based on similar frequency-specific ReHo signature. The forging studies indicated that analyzing time-frequency characteristics is equally important for comprehensively exploring how different brain systems/sub-systems coordinate.

One challenge of further extracting the time-frequency characteristic in frequency clustering analysis is time-frequency representation of fMRI signals. In many previous studies, the time-frequency characteristics of fMRI time series were usually measured using short-time Fourier transform (Mezer et al., 2009) or wavelet transform (Bullmore et al., 2001; Shimizu et al., 2004) which always assume the linearity or stationarity of input signals (Huang and Shen, 2005). However, BOLD time series may not conform to these assumptions (Lange and Zeger, 1997). Furthermore, constrained by the Uncertainty Principle (Robertson, 1929), most of the traditional time-frequency methods are limited in providing both high temporal resolution and high frequency resolution at the same time.

HHT is a novel time-frequency method suitable for both non-linear and non-stationary signals. Its application to electrophysiological studies has demonstrated its efficay in providing fine expressions of instantaneous frequency (Huang and Shen, 2005; Peng et al., 2005; Donnelly, 2006; Huang and Wu, 2008; Huang et al., 2008). For example, HHT has been successfully applied in EEG-based seizure classification (Oweis and Abdulhay, 2011), detection of spindles in sleep EEGs (Yang et al., 2006), and ECG de-noising (Tang et al., 2007). However, its application in fMRI studies is rare.

Other challenges in time-frequency analysis based frequency clustering analysis voxel-wised analysis at different frequency bands may demand great amount of calculation. Furthermore, in the stage of clustering analysis, the labels of the clusters change randomly across analyses (Mezer et al., 2009), causing difficulty in cross-condition/datasets comparisons.

In this article, we introduced a novel frequency clustering analysis method based on HHT and an improved k-mean clustering method using label-replacement procedure. In our framework, first, the time series from multiple predefined regions of interest (ROIs) [i.e., 90 ROIs defined by the Automated Anatomical Labeling (AAL) template Tzourio-Mazoyer et al., 2002] were extracted. Second, each time series was decomposed into several intrinsic mode functions (IMFs) of which the instantaneous frequency characteristics were subsequently calculated using HHT. Third, the improved k-means clustering method using a label-replacement method was applied to the data of each subject to classify the ROIs into different classes. To test the efficacy of our frequency clustering analysis method, two independent resting-state fMRI data sets of healthy subjects (198 subjects in Dataset I; 88 subjects in Dataset II) were analyzed. The results demonstrated that for different dataset, our method generated stable clusters of the brain regions according to time-frequency characteristics of their resting state BOLD activities.

## Materials and methods

### fMRI data acquisition

In this study, we used a resting-state fMRI dataset (Dataset I) provided by the open source website of “1,000 Functional Connectomes' Project” (http://www.nitrc.org/projects/fcon_1000/). The dataset included functional and structural MR images recorded from 198 healthy subjects (18–26 years old, 122 females) by Yu-Feng Zang (Song et al., 2014). No subject had a history of neurological, psychiatric or medical conditions. Written informed consents were given to all subjects in accordance with Institutional Review Board guidelines and in compliance with the Declaration of Helsinki.

The scanning was performed using a 3.0-Tesla scanner (Siemens TRIO TIM, Munich, Germany). The subjects were instructed to rest with their eyes closed, keep their heads still, and not to fall asleep. A gradient echo T2^*^-weighted EPI sequence was used for acquiring resting state functional images with the following parameters: TR = 2,000 ms; TE = 30 ms; 33 slices; matrix size = 64 × 64; FOV = 240 × 240 mm^2^ acquisition voxel size = 3.75 × 3.75 × 3.50 mm^3^; 225 volumes.

In order to verify the stability of the results, an independent resting-state data set (Dataset II) was also analyzed. Dataset II was collected from 88 healthy young right-handed college students (19–26 years old, 44 females) performing eyes-closed resting state task. The scanning was performed using a 3.0-Tesla Siemens whole-body MRI system in Brain Imaging Center of Beijing Normal University. All subjects were given the written informed consents before scanning. No subject had a history of neurological, psychiatric, or medical conditions. The scan was performed during a resting-state condition. The detailed parameters used were as follows: TR = 2,000 ms; TE = 30 ms; 33 slices; matrix size = 64 × 64; FOV = 240 × 240 mm^2^ acquisition voxel size = 3.13 × 3.13 × 3.60 mm^3^; 145 volumes. The experiment was approved by the Institutional Review Board of the Beijing Normal University.

### Image preprocessing

In current research, the images were analyzed using SPM8 (http://www.fil.ion.ucl.ac.uk/spm). For each subject, the original first five functional volumes were removed to avoid the possible disturbance caused by non-equilibrium effects of magnetization. The remaining functional images (220 in Dataset I; 140 in Dataset II) were corrected for slice timing, motion corrected, and spatially normalized into a Montreal Neurological Institute (MNI) space using the standard EPI template (Evans et al., 1993). The normalized image had 61 slices, a matrix size of 61 × 73, and a voxel size of 3 mm × 3 mm × 3 mm. No translation or rotation movement in any data set exceeded ±2 mm or ±2 degree. The data had originally been “cleaned” through the use of confound regressors derived from CSF and white matter masks, as well as head motion parameters. The linear trend was regressed out for each voxel's time course to remove signal drifts caused by scanner instability or other factors. The time course of each voxel was normalized by subtracting the temporal mean and dividing by the temporal standard deviation. After preprocessing, for each subject and for each of the 90 regions of interest (ROIs) defined using the AAL template (Tzourio-Mazoyer et al., 2002), an ROI time course was extracted by averaging the time courses of all voxels in the ROI.

### Using Hilbert-Huang transform (HHT) to acquire instantaneous frequency and Hilbert weighted frequency (HWF)

The forgoing ROI time courses were fed into HHT to acquire instantaneous frequency and HWF feature. The HHT consists of three main processes. First, major IMFs are extracted from the input signal based on empirical mode decomposition (EMD). Second, Hilbert transform is applied to each IMF to obtain the analytic transform of the original signal. Last, the instantaneous frequency is calculated according to the analytical transform of each IMF (Huang and Shen, 2005; Huang and Pan, 2006; Ding et al., 2007) and the Hilbert weighted frequency (Xie and Wang, 2006) of each IMF is calculated according to the instantaneous frequency of the IMF. The detailed descriptions of each step are as follows:

(1) Empirical mode decomposition

The EMD method (Huang and Shen, 2005) decomposes an input signal into a finite set of intrinsic oscillatory components, namely, the IMFs. Mathematically, for fMRI time series, EMD generates a set of IMFs and a monotonic residue signal *r*(*t*):

(1) $$\begin{array}{l} x\left(t\right)=\overset{N}{\underset{i=1}{\sum }}IMF_{i}\left(t\right)\;+\;r\left(t\right), \end{array}$$

where N is the number of the IMFs.

Each IMF must satisfy two conditions:

- Along the time course of the IMF, the number of the local extrema and the number of zero crossings are either equal or differ by one;
- The sum of the envelope defined by the local maxima and the envelope defined by the local minima is constantly zero.

To extract IMFs using EMD, an iterative method known as the sifting algorithm is used as follows:

Step 1: Find the local extrema of the input signal;
Step 2: Use interpolation to generate the lower envelope *elow*(*t*) and the upper envelope *eup*(*t*) of the current signal according to the local minima and local maxima respectively;
Step 3: Calculate the local mean time course *emean*(*t*):
  (2) $$\begin{array}{l} emean\left(t\right)\;=\;\frac{eup\left(t\right)\;+\;elower\left(t\right)}{2}, \end{array}$$
Step 4: Obtain the “oscillatory-mode” *r*(*t*) = *x*(*t*) − *emean*(*t*);
Step 5: If *r*(*t*) satisfies the stopping criteria (the two conditions of IMF), *IMF*_*i*_ = *r*(*t*) becomes an IMF, otherwise set *x*(*t*) = *r*(*t*) and repeat the process from Step 1.

To obtain remaining IMFs, the same procedure is applied iteratively to the residual *r*(*t*) = *x*(*t*)−*IMF*_*i*_(*t*) until *r(t)* is monotonic.

(2) Extracting instantaneous frequency using Hilbert transform.

Hilbert transform was used to extract the instantaneous frequency of each IMF. For signal *x*(*t*), its Hilbert transform *y*(*t*) is defined as:

(3) $$\begin{array}{l} y\left(t\right)=\frac{P}{\pi }\overset{+\infty }{\underset{-\infty }{\int }}\frac{x\left(\tau \right)}{t-\tau }d\tau , \end{array}$$

where *P* is the Cauchy principal value (Surhone et al., 2013). Hilbert transform is capable of describe the local properties of *x*(*t*) (Peng et al., 2005). The analytic transform of *z*(*t*) *x*(*t*) is defined as:

(4) $$\begin{array}{r} z\left(t\right)=x\left(t\right)\;+\;iy\left(t\right)=a\left(t\right)e^{i\varphi \left(t\right)}, \end{array}$$

(5) $$\begin{array}{r} a\left(t\right)=\sqrt{\left[x^{2}\left(t\right)\;+\;y^{2}\left(t\right)\right]}, \end{array}$$

(6) $$\begin{array}{r} \varphi \left(t\right)=\text{arctan }\left(\frac{y\left(t\right)}{x\left(t\right)}\right), \end{array}$$

where *a*(*t*) is the instantaneous amplitude, and φ(*t*) is the instantaneous phase. The instantaneous frequency ω(*t*) is defined as the time derivative of φ(*t*):

(7) $$\begin{array}{l} \omega \left(t\right)=\frac{d\varphi \left(t\right)}{dt}. \end{array}$$

(3) Hilbert weighted frequency (HWF) based on instantaneous frequency

The Hilbert weighted frequency (Xie and Wang, 2006) of each IMF is also calculated based on the instantaneous amplitude and phase to reflect the mean oscillation frequency of the IMF. The *HWF*(*j*) of the jth IMF is defined as:

(8) $$\begin{array}{l} HWF\left(j\right)=\frac{\overset{m}{\underset{i=1}{\sum }}\omega_{j}\left(i\right)a_{j}^{2}\left(i\right)}{\overset{m}{\underset{i=1}{\sum }}a_{j}^{2}\left(i\right)}, \end{array}$$

where ω_*j*_(*i*) is the instantaneous frequency, *a*_*j*_(*t*) is the instantaneous amplitude, and *m* is the number of time point.

### Identify the brain networks using k-means clustering analysis based on HWF characteristics

In order to identify the brain regions sharing common instantaneous frequency characteristics, we employed k-means clustering analysis to the two resting-state datasets respectively. In each analysis, the 90 ROIs were classified into different clusters based on the HWF feature vectors each of which comprised the first five HWFs. For each feature vector, each HWF form all subjects was concatenated to yield a group feature vector for the following analysis. The clustering analysis was performed for different k from 1 to 90. The squared Euclidean distance index (Mezer et al., 2009) for different k values was plotted as a function to determine the appropriate k.

### Label-replacement method to improve k-means clustering analysis

In conventional k-means clustering analysis, the labels of each cluster are unordered due to random initialization of the algorithm which hinders the compare between conditions and datasets. The first impede caused by the randomization is that the label of a cluster, of which the spatial structure changes little, may change dramatically from run to run even for the same condition in the same dataset. For example, some brain regions are classified into a cluster labeled as “1” in one run, and into the same cluster but labeled as “2” in another run. In this study, we proposed a method for sorting the label of clusters. The method composed of two steps: (1) label-sorting and (2) label-matching. In label-sorting, a hash table (Maurer and Lewis, 1975) was used to improve the computing efficiency. The detailed process was as follows:

Step 1: Obtain an unordered label table (*ULT*(*i*)) according to the raw results of k-means clustering.
Step 2: A hash table (*Hash*(*i*)) is constructed to record the labels in turn.
Step 3: If the label is not found in *Hash*(*i*); add the label to *Hash*(*i*) and record the order by *Lab* If the label already exists in *Hash*(*i*); replace the label of *ULT*(*i*) by *Lab*; update *Lab* and return to Step2.

**Figure 1:**
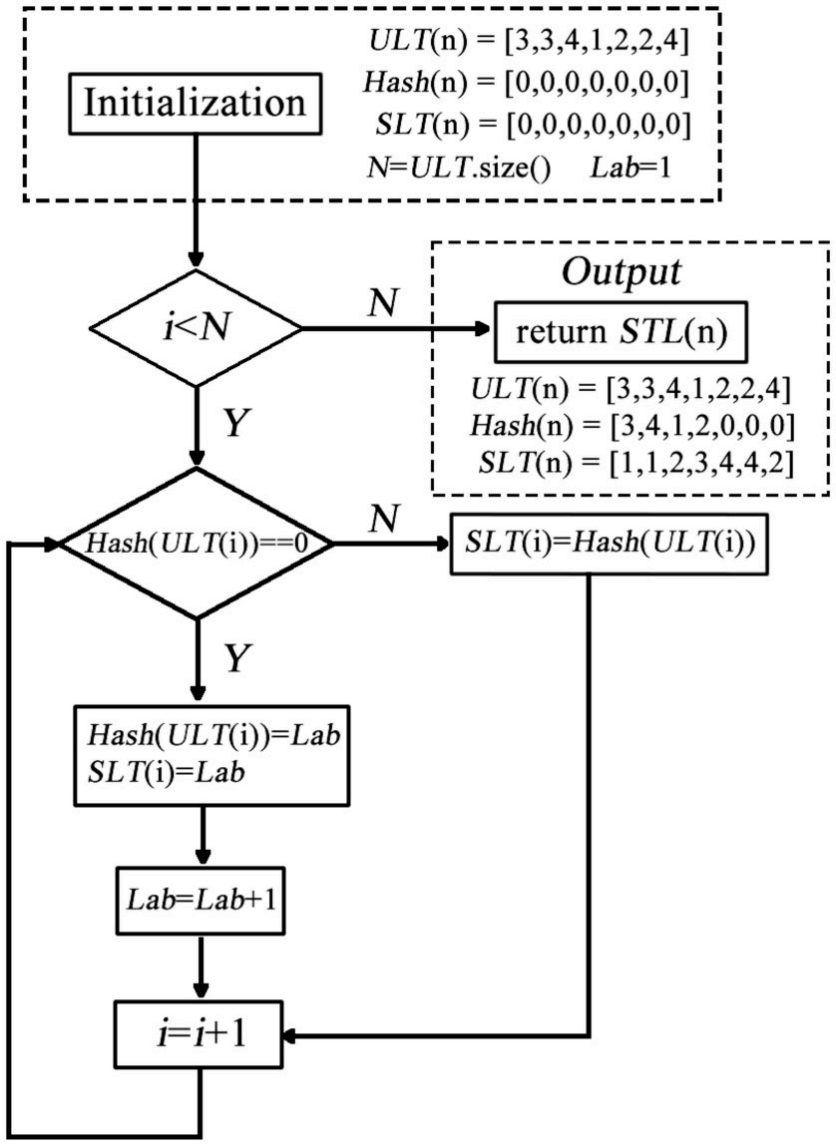
**The process of label-sorting algorithm**.

A sorted label table (*SLT*(*n*)) is constructed by the follow process (shown in Figure 1):

In this part, the hash table is used to record a new label of each *ULT*(*n*). In each repetition, the algorithm searches the hash table to decide whether the unordered label needs to be replaced. After label-sorting, the *ULT*(*i*) was sorted in the order of brain regions (AAL).

The second impede caused by the randomization is that label may change across different conditions/datasets. That is, for the same cluster, its label within one condition/dataset could be different from that in another condition/dataset, making it hard to compare between different groups or between different conditions. This could be even worse when the cluster changes slightly across conditions/dataset. Therefore, we developed a label-matching method besides the forgoing label sorting method. The basic idea is that use the label setting of one of the condition/dataset as a reference, then go through the target clusters of another condition/dataset, when a target cluster share a similar spatial pattern with a reference cluster, the label of this reference cluster is assigned to the target cluster.

**Figure 2:**
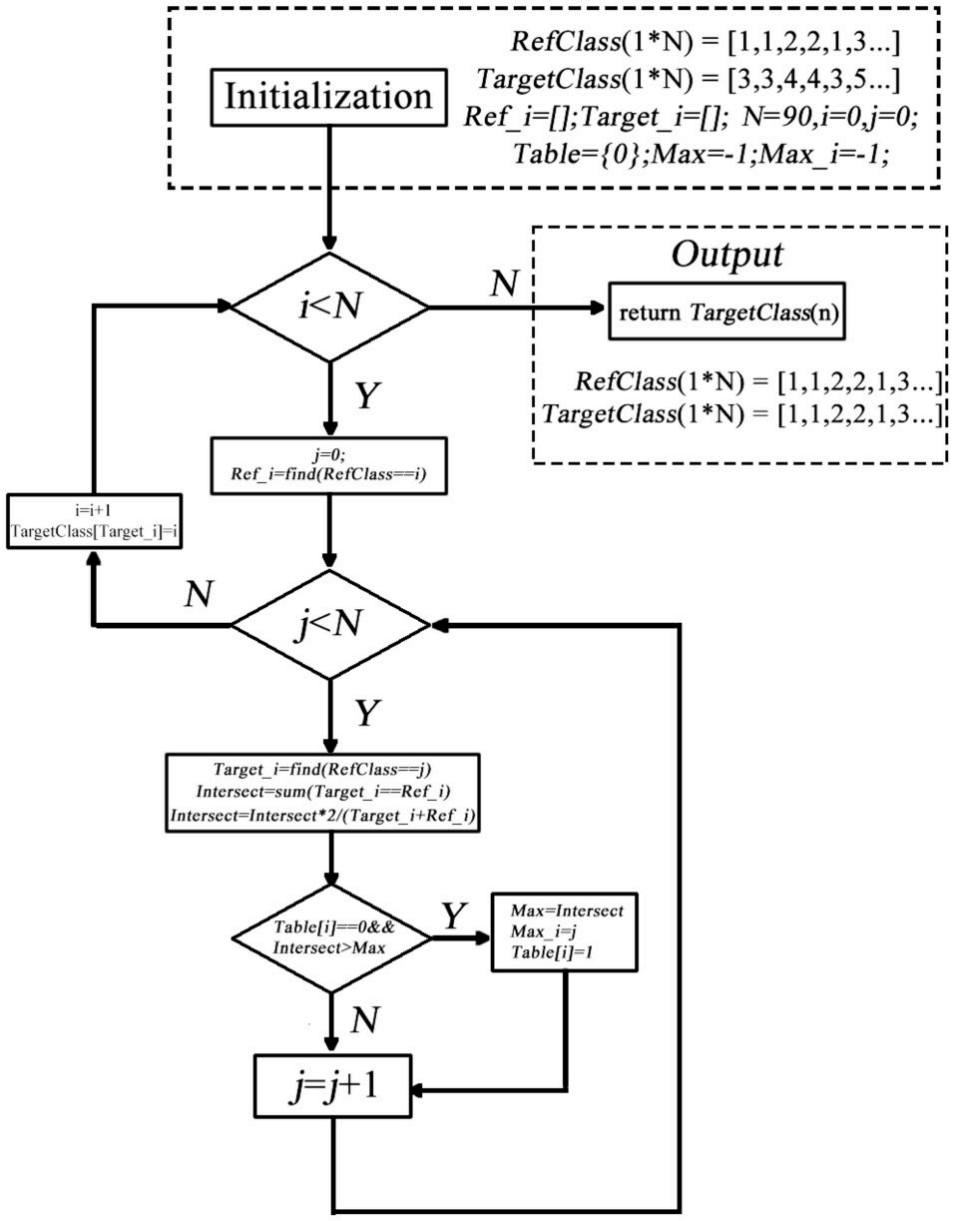
**The process of label-matching algorithm**.

The detail of matching the target and the reference cluster is as below (shown in Figure 2).

## Results

**Figure 3:**
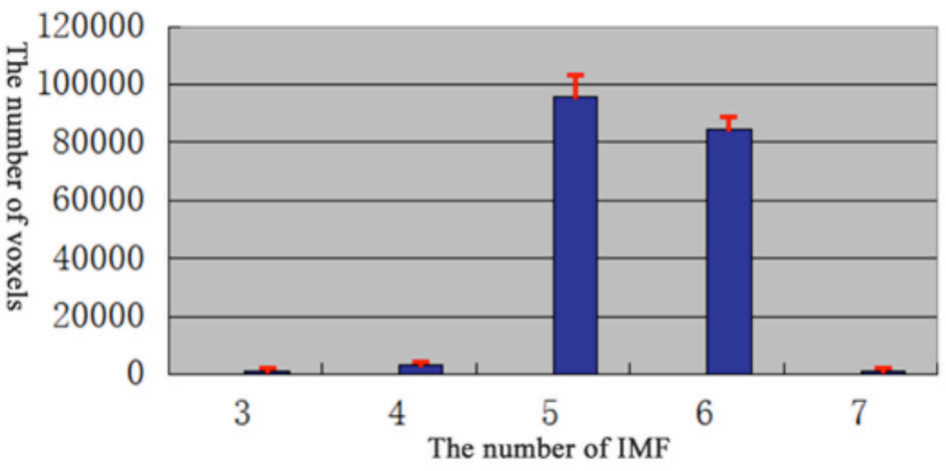
**Histogram of the number of IMF**.

For each dataset, BOLD time series from the 90 ROIs defined by AAL template were extracted. First, EMD was applied to decompose the BOLD time series into different frequency components. The EMD outcome of both datasets showed that the BOLD signals could be decomposed into five major IMFs (please see examples in Figure 3).

In order to determine the number of IMFs which were self-adaptive decomposed from fMRI signal, the EMD of each vowel in each subjects were calculated.

**Figure 4:**
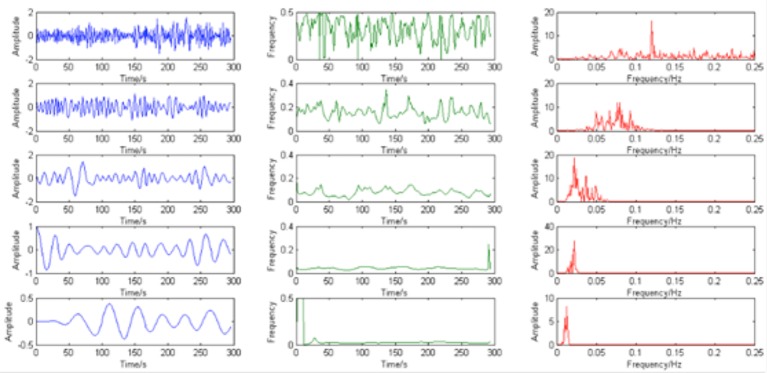
**The example of IMFs (blue), instantaneous frequency (green) and corresponding power spectrum (red)**.

According to the results (Figure 3), most of the voxels have at least 5 IMFs. Therefore, in our study, the first 5 IMFs were selected for the following analysis (an example of EMD was shown in Figure 4, left panel).

Second, the instantaneous frequency of each major IMF was calculated using Hilbert transform and its corresponding power spectrum was calculated. The results demonstrate that the instantaneous frequency of the IMFs varies across frequency bands centered by different dominant frequency from low (around 0.01 Hz) to high (around 0.12 Hz) (please see the example in Figure 4).

**Figure 5:**
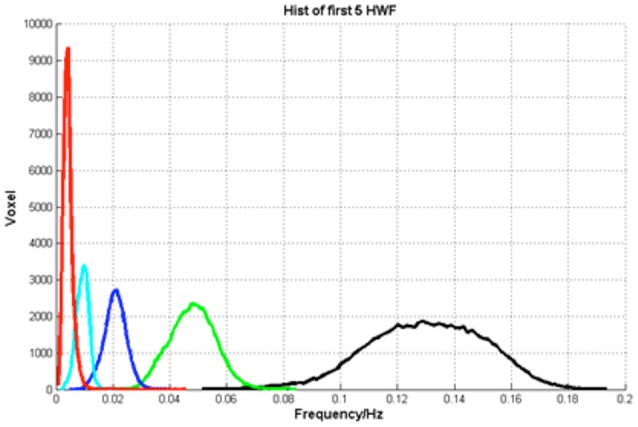
**The histograms of HWF**.

Third, the HWF of each major IMF was calculated. The histograms of HWF distributions in the whole brain across all subjects showed that the major IMFs occupy certain frequency bands: IMF1, 0–0.01 Hz; IMF2, 0.005–0.015 Hz; IMF3 0.01–0.03 Hz; IMF4, 0.03–0.07 Hz; and IMF5, 0.08–0.18 Hz (Figure 5).

**Figure 6:**
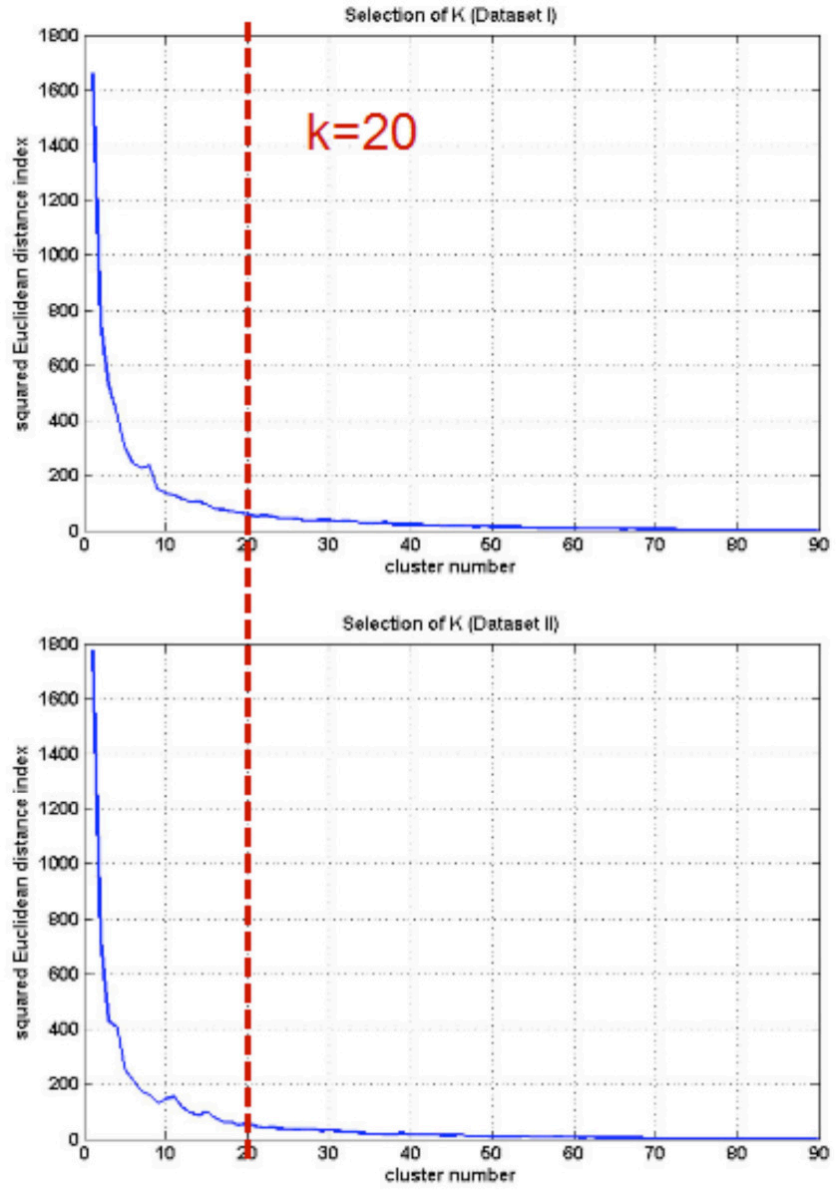
**Selection of cluster number (k)**.

The first five HWF of each ROI were selected as the features for k-means clustering analysis. For selecting the appropriate parameter (k), k-means clustering analysis was repeated for different k from 1 to 90 (shown in Figure 6). Then the clusters were evaluated using a squared Euclidean distance index (Mezer et al., 2009) and the appropriate parameter, *k* = 20, was selected for each subject.

**Figure 7:**
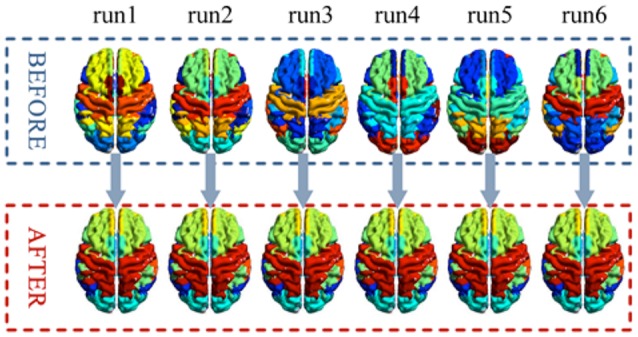
**A comparison of the stability of before and after label-sorting method in each run**.

A comparison of the stability of before and after label-sorting method was shown in Figure 7. After label-sorting, the results of cluster were sorted in the order of brain regions.

**Figure 8:**
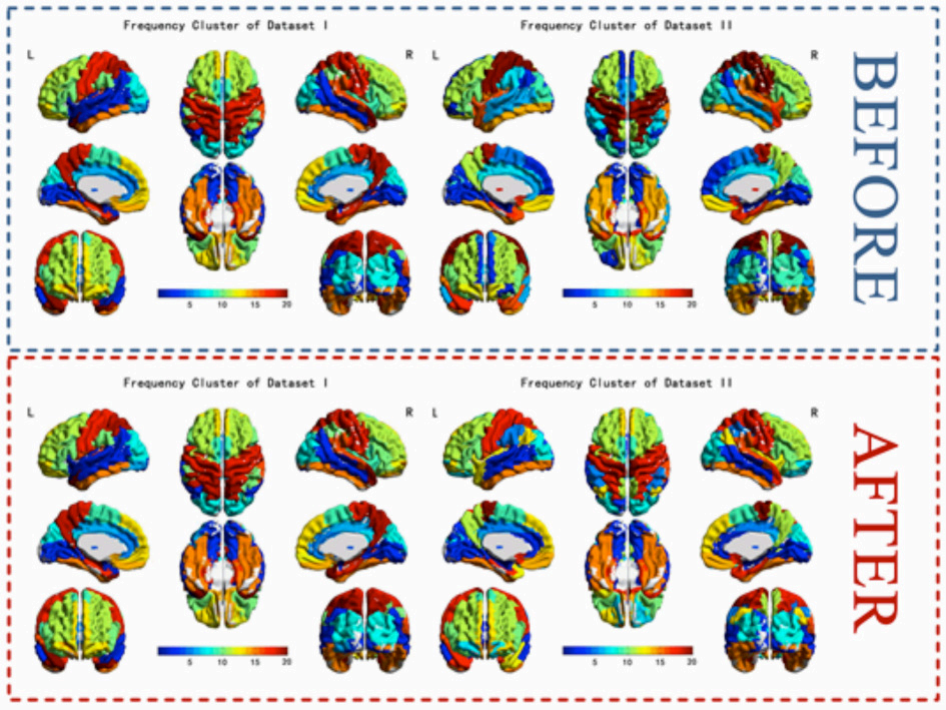
**The k-means frequency clustering analysis of two independent fMRI data sets and a comparison of clusters of before and after label-replacement**.

The results of the two datasets show almost identical clusters of the ROIs and a comparison of the results of before and after label-replacement (shown in Figure 8).

## Discussion

In this study, we presented a new frame work for brain region clustering based on HHT method and an improved k-mean clustering method adopting cluster label sorting, and applied the frame work to analyzing two independent resting-state fMRI data sets of healthy subjects (198 subjects in Dataset I; 88 subjects in Dataset II). The results showed that our method is efficacy in functional segregation based on time-frequency characteristics of resting state BOLD activities.

### Applying Hilbert-Huang transform to BOLD signal analysis

In our study, HHT was introduced to frequency clustering analysis of resting-state fMRI. The advantage of using HHT is mainly based on two features of the fMRI data. First, previous studies have shown that the fMRI data may not strictly conform to the assumptions of linearity and stationarity (Lange and Zeger, 1997). Compared with traditional time-frequency analysis method such as wavelet transform, short-time Fourier transform, HHT can be directly applied to the non-linear or non-stationary signals. Second, the fMRI signals mainly record the slow BOLD change in low sampling intervals around 1–3 s. Traditional time-frequency methods constrained by the Uncertainty Principle (Robertson, 1929) are limited in providing both high temporal resolution and high frequency resolution at the same time. While many previous studies have shown that HHT does not suffer from the trade-off between frequency resolution and temporal resolution (Peng et al., 2005; Donnelly, 2006; Huang and Wu, 2008) and thus may be a suitable candidate for time-frequency representation of the fMRI signals. Our results show that HHT can represent the fMRI signals in both high temporal resolution and high frequency resolution.

### Number of IMFs in empirical mode decomposition

EMD method, as an important part of HHT, is a local, fully data driven and self-adaptive analysis approach. The results of EMD show that different brain voxel/regions may contain multiple IMFs, the number of IMF voxels may affect the efficacy of clustering in the features space. On one hand, too small number of IMFs may quicken the processing but provides inadequate information to describe the functional significance of the clusters. On the other hand, too larger number of IMFs may impede the clustering progress. In our method, the number of IMFs was determined by picking up the number that present in most of the brain voxels (Figure 3). The HWF of each IMF was determined using instantaneous amplitude and phase information to reflect the mean oscillation frequency of the IMF. Previous research has shown that HWF has clear physical meaning and has low variability in terms of robustness (Xie and Wang, 2006). Therefore, the HWF of the first five IMFs were calculated to construct the feature vector. And then, the histogram of HWF was also calculated (shown in Figure 5) to show the mean frequency distribution of the five IMFs. Each of the five histograms is a statistic of the whole-brain voxels. Since the frequency content of different voxels at different sites of the brain (and subjects) are roughly similar, the same IMF (*IMF*(*j*), *j* = 1, 2, 3, 4, 5) from any voxel will roughly fall into the same frequency band. These results suggest that EMD works well in adaptively decomposing the fMRI signals into different IMFs that fall into distinctive frequency bands and is a promising method for non-stationary and non-linear neurological signal processing.

### Label-sorting for k-means clustering

In previous frequency clustering analysis, k-means clustering method has been applied to resting-state fMRI network analysis (Song et al., 2014) and generates meaningful results. However, in the previous work, the labels of the clusters were randomly assigned and changed from analysis to analysis, making it hard to compare between conditions/datasets. Our study presents a label-sorting method which uses Hash table to obtain an ordered and stable clustering result across different runs of analysis within a condition/dataset, and further a cluster-label matching method to deal with cluster matching and label assignment across conditions/datasets. The verification results showed almost identical clusters no matter when the method was applied to different runs of a dataset or to different datasets, indicating a stable performance of our framework (Figures 7, 8). It is worth noting that when condition or dataset changes, the spatial representation of an underlying brain cluster may also change in some extends according to the real scenario. A careful visual inspection for potential unmatched cluster and label caused by dramatic brain change between conditions or subject groups is recommended besides our frame work. The change itself, if significant, could deliver meaningful clinical, neurological and psychophysiological information.

### Selecting the regions of interest

It is worth noting that using AAL template or for ROI selection is not part of the major line but an alternative module of our frame work. In the current study we used the classical AAL template for ROI selection to demonstrate the performance of our method. However, AAL template is defined anatomically. The current work only clustered the known anatomical structures function similarly on frequency domain. Indeed, the AAL ROI template can be replaced by other ROIs or voxels according to the interest of the researchers. The ROIs can be a set of task-activated sites with their intrinsic relationship to be clarified, or a set of anatomically defined structures. The analysis can also be performed in a whole-brain or partial-brain voxel-wise fashion.

### Limitation of the current work

In the current work, the data driven process introduce five IMFs referring to different frequency bands. The IMF 5 was corresponding to a frequency band of 0.08~0.18 Hz which had been excluded in most of the previous resting state functional connectivity analysis. It is a nontrivial question that what information the higher frequency bands of BOLD change provide. Although, faster neural electrophysiological activities have been found in higher order regions such as the frontal lobe and were proposed to carry important cognitive meanings (Lang et al., 1986), the neurocognitive meaning of the higher frequency components of BOLD which were usually considered as noise in many previous functional connectivity studies is under debate (Michels et al., 2010; Boubela et al., 2013). Understanding the neurocognitive meanings of the clustering results requires further careful works in the future.

## Conclusion

In this study, a novel frequency clustering analysis method based on HHT and a label-replacement procedure was introduced. First, the ROI time series were extracted. Second, each time series was decomposed into several intrinsic mode functions (IMFs) by using HHT. Third, the improved k-means clustering method using a label-replacement method was applied to the data of each subject to classify the ROIs into different classes. Two independent resting-state fMRI dataset of healthy subjects were analyzed to test the efficacy of method. The results showed that for different dataset, our method can stably cluster the brain regions according to the time-frequency characteristics of their resting state BOLD activities.

## Author contributions

XWe, method instruction. TW, algorithm development. LY, method instruction. XWu, method instruction. CL, method instruction.

### Conflict of interest statement

The authors declare that the research was conducted in the absence of any commercial or financial relationships that could be construed as a potential conflict of interest.

## References

[B1] Boubela R. N. Kalcher K. Huf W. Kronnerwetter C. Filzmoser P. Moser E. (2013). Beyond noise: using temporal ICA to extract meaningful information from high-frequency fMRI signal fluctuations during rest. Front. Hum. Neurosci. 7:168. 10.3389/fnhum.2013.00168 23641208 PMC3640215

[B2] Bullmore E. Long C. Suckling J. Fadili J. Calvert G. Zelaya F.. (2001). Colored noise and computational inference in neurophysiological (fMRI) time series analysis: resampling methods in time and wavelet domains. Hum. Brain Mapp. 12, 61–78. 10.1016/S1053-8119(01)91429-6 11169871 PMC6871881

[B3] Calhoun V. D. Liu J. Adali T. (2009). A review of group ICA for fMRI data and ICA for joint inference of imaging, genetic, and ERP data. Neuroimage 45, S163–S172. 10.1016/j.neuroimage.2008.10.057 19059344 PMC2651152

[B4] Damoiseaux J. S. Rombouts S. A. R. B. Barkhof F. Scheltens P. Stam C. J. Smith S. M.. (2006). Consistent resting-state networks across healthy subjects. Proc. Natl. Acad. Sci. U.S.A. 103, 13848–13853. 10.1073/pnas.0601417103 16945915 PMC1564249

[B5] De Luca M. Beckmann C. F. De Stefano N. Matthews P. M. Smith S. M. (2006). fMRI resting state networks define distinct modes of long-distance interactions in the human brain. Neuroimage 29, 1359–1367. 10.1016/j.neuroimage.2005.08.035 16260155

[B6] Ding H. Huang Z. Song Z. Yan Y. (2007). Hilbert–Huang transform based signal analysis for the characterization of gas–liquid two-phase flow. Flow Meas. Instrum. 18, 37–46. 10.1016/j.flowmeasinst.2006.12.004

[B7] Donnelly D. (2006). The fast Fourier and Hilbert-Huang transforms: a comparison, in The Proceedings of the Multiconference on Computational Engineering in Systems Applications (Beijing), 84–88.

[B8] Evans A. C. Collins D. L. Mills S. R. Brown E. D. Kelly R. L. Peters T. M. (1993). 3D statistical neuroanatomical models from 305 MRI volumes, in 1993 IEEE Conference Record Nuclear Science Symposium and Medical Imaging Conference (San Francisco, CA), 1813–1817.

[B9] Fox P. T. Laird A. R. Lancaster J. L. (2005). Coordinate-based voxel-wise meta-analysis: dividends of spatial normalization. Report of a virtual workshop. Hum. Brain Mapp. 25, 1–5. 10.1002/hbm.20139 15846826 PMC6871692

[B10] Fransson P. Marrelec G. (2008). The precuneus/posterior cingulate cortex plays a pivotal role in the default mode network: evidence from a partial correlation network analysis. Neuroimage 42, 1178–1184. 10.1016/j.neuroimage.2008.05.059 18598773

[B11] Huang H. Pan J. (2006). Speech pitch determination based on Hilbert-Huang transform. Signal Process. 86, 792–803. 10.1016/j.sigpro.2005.06.011

[B12] Huang M. Wu P. Liu Y. Bi L. Chen H. (2008). Application and contrast in brain-computer interface between hilbert-huang transform and wavelet transform, in 2008 The 9th International Conference for Young Computer Scientists (Hunan), 1706–1710.

[B13] Huang N. E. Shen S. S. P. (2005). Hilbert-Huang Transform and Its Applications. Singapore: World Scientific 10.1142/5862

[B14] Huang N. E. Wu Z. (2008). A review on Hilbert-Huang transform: method and its applications to geophysical studies. Rev. Geophys. 46:RG2006 10.1029/2007RG000228

[B15] Lang W. Lang M. Kornhuber A. Kornhuber H. H. (1986). Electrophysiological evidence for right frontal lobe dominance in spatial visuomotor learning. Arch. Ital. Biol. 124, 1–13. 3741032

[B16] Lange N. Zeger S. L. (1997). Non-linear fourier time series analysis for human brain mapping by functional magnetic resonance imaging. J. R. Stat. Soc. Ser. C 46, 1–29. 10.1111/1467-9876.00046

[B17] Li R. Chen K. Fleisher A. S. Reiman E. M. Yao L. Wu X. (2011). Large-scale directional connections among multi resting-state neural networks in human brain: a functional MRI and Bayesian network modeling study. Neuroimage 56, 1035–1042. 10.1016/j.neuroimage.2011.03.010 21396456 PMC3319766

[B18] Lowe M. J. (2010). A historical perspective on the evolution of resting-state functional connectivity with MRI. Magn. Reson. Mater. Phys. Biol. Med. 23, 279–288. 21076991 10.1007/s10334-010-0230-y

[B19] Maurer W. D. Lewis T. G. (1975). Hash table methods. ACM Comput. Surv. 7, 5–19. 10.1145/356643.356645

[B20] Mezer A. Yovel Y. Pasternak O. Gorfine T. Assaf Y. (2009). Cluster analysis of resting-state fMRI time series. Neuroimage 45, 1117–1125. 10.1016/j.neuroimage.2008.12.015 19146962

[B21] Michels L. Bucher K. Lüchinger R. Klaver P. Martin E. Jeanmonod D.. (2010). Simultaneous EEG-fMRI during a working memory task: modulations in low and high frequency bands. PLoS ONE 5:e10298. 10.1371/journal.pone.0010298 20421978 PMC2858659

[B22] Oweis R. J. Abdulhay E. W. (2011). Seizure classification in EEG signals utilizing Hilbert-Huang transform. Biomed. Eng. Online 10, 38–52. 10.1186/1475-925X-10-38 21609459 PMC3116477

[B23] Peng Z. K. Peter W. T. Chu F. L. (2005). A comparison study of improved Hilbert–Huang transform and wavelet transform: application to fault diagnosis for rolling bearing. Mech. Syst. Signal Process. 19, 974–988. 10.1016/j.ymssp.2004.01.006

[B24] Raichle M. E. MacLeod A. M. Snyder A. Z. Powers W. J. Gusnard D. A. Shulman G. L. (2001). A default mode of brain function. Proc. Natl. Acad. Sci. U.S.A. 98, 676–682. 10.1073/pnas.98.2.676 11209064 PMC14647

[B25] Robertson H. P. (1929). The uncertainty principle. Phys. Rev. 34:163 10.1103/physrev.34.163

[B26] Salvador R. Suckling J. Schwarzbauer C. Bullmore E. (2005). Undirected graphs of frequency-dependent functional connectivity in whole brain networks. Philos. Trans. R. Soc. Lond. B Biol. Sci. 360, 937–946. 10.1098/rstb.2005.1645 16087438 PMC1854928

[B27] Shimizu Y. Barth M. Windischberger C. Moser E. Thurner S. (2004). Wavelet-based multifractal analysis of fMRI time series. Neuroimage 22, 1195–1202. 10.1016/j.neuroimage.2004.03.007 15219591

[B28] Song X. Zhang Y. Liu Y. (2014). Frequency specificity of regional homogeneity in the resting-state human brain. PLoS ONE 9:e86818. 10.1371/journal.pone.0086818 24466256 PMC3900644

[B29] Sun F. T. Miller L. M. D'Esposito M. (2005). Measuring temporal dynamics of functional networks using phase spectrum of fMRI data. Neuroimage 28, 227–237. 10.1016/j.neuroimage.2005.05.043 16019230

[B30] Surhone L. M. Tennoe M. T. Henssonow S. F. Cauchy A. L. (2013). Cauchy Principal Value. Betascript Publishing.

[B31] Tang J. T. Zou Q. Tang Y. Liu B. Zhang X.-K. (2007). Hilbert-Huang transform for ECG de-noising, in 2007 1st International Conference on Bioinformatics and Biomedical Engineering (Wuhan), 664–667.

[B32] Tzourio-Mazoyer N. Landeau B. Papathanassiou D. Crivello F. Etard O. Delcroix N.. (2002). Automated anatomical labeling of activations in SPM using a macroscopic anatomical parcellation of the MNI MRI single-subject brain. Neuroimage 15, 273–289. 10.1006/nimg.2001.0978 11771995

[B33] Van Den Heuvel M. P. Pol H. E. H. (2010). Exploring the brain network: a review on resting-state fMRI functional connectivity. Eur. Neuropsychopharmacol. 20, 519–534. 10.1016/j.euroneuro.2010.03.008 20471808

[B34] Wei L. Duan X. Zheng C. Wang S. Gao Q. Zhang Z.. (2014). Specific frequency bands of amplitude low-frequency oscillation encodes personality. Hum. Brain Mapp. 35, 331–339. 10.1002/hbm.22176 22987723 PMC6869309

[B35] Wen X. Mo J. Ding M. (2012). Exploring resting-state functional connectivity with total interdependence. Neuroimage 60, 1587–1595. 10.1016/j.neuroimage.2012.01.079 22289806 PMC3516187

[B36] Wu X. Li R. Fleisher A. S. Reiman E. M. Guan X. Zhang Y.. (2011). Altered default mode network connectivity in Alzheimer's disease—a resting functional MRI and Bayesian network study. Hum. Brain Mapp. 32, 1868–1881. 10.1002/hbm.21153 21259382 PMC3208821

[B37] Xie H. Wang Z. (2006). Mean frequency derived via Hilbert-Huang transform with application to fatigue EMG signal analysis. Comput. Methods Programs Biomed. 82, 114–120. 10.1016/j.cmpb.2006.02.009 16616796

[B38] Yang H. Long X.-Y. Yang Y. Yan H. Zhu C.-Z. Zhou X.-P.. (2007). Amplitude of low frequency fluctuation within visual areas revealed by resting-state functional MRI. Neuroimage 36, 144–152. 10.1016/j.neuroimage.2007.01.054 17434757

[B39] Yang Z. Yang L. Qi D. (2006). Detection of spindles in sleep EEGs using a novel algorithm based on the Hilbert-Huang transform. Wavelet Anal. Appl. 543–559. 10.1007/978-3-7643-7778-6_40

[B40] Yu R. Hsieh M. H. Wang H. L. S. Liu C. M. Liu C. C. Hwang T. J.. (2013). Frequency dependent alterations in regional homogeneity of baseline brain activity in schizophrenia. PLoS ONE 8:e57516. 10.1371/journal.pone.0057516 23483911 PMC3590274

[B41] Zang Y. Jiang T. Lu Y. He Y. Tian L. (2004). Regional homogeneity approach to fMRI data analysis. Neuroimage 22, 394–400. 10.1016/j.neuroimage.2003.12.030 15110032

